# Chikungunya Virus Infection during Pregnancy, Réunion, France, 2006

**DOI:** 10.3201/eid1604.091403

**Published:** 2010-03

**Authors:** Xavier Fritel, Olivier Rollot, Patrick Gérardin, Bernard-Alex Gaüzère, Jacques Bideault, Louis Lagarde, Barbara Dhuime, Eric Orvain, Fabrice Cuillier, Duksha Ramful, Sylvain Sampériz, Marie-Christine Jaffar-Bandjee, Alain Michault, Liliane Cotte, Monique Kaminski, Alain Fourmaintraux, the Chikungunya-Mère-Enfant Team

**Affiliations:** Centre Hospitalier Régional de la Réunion, Saint-Denis, France (X. Fritel, P. Gérardin, B.-A. Gaüzère, E. Orvain, F. Cuillier, D. Ramful, S. Sampériz, M.-C. Jaffar-Bandjee, A. Michault, L. Cotte, A. Fourmaintraux); Centre d’Investigation Clinique–Epidémiologie Clinique de la Réunion, Saint-Denis (O. Rollot, P. Gérardin); Institut National de la Santé et de la Recherche Médicale, Villejuif, France (X. Fritel, P. Gérardin, M. Kaminski); Centre Hospitalier Intercommunal de Saint-Benoit-Saint-André, Saint-Benoit, France (J. Bideault); Centre Hospitalier Gabriel-Martin, Saint-Paul, France (L. Lagarde); Clinique Sainte-Clotilde, Saint-Denis (B. Dhuime); Université Pierre et Marie Curie 6, Paris, France (X. Fritel, P. Gérardin, M. Kaminski)

**Keywords:** Chikungunya virus, pregnancy, mothers, newborns, infectious disease transmission, alphavirus infections, disease outbreaks, viruses, Réunion, research

## Abstract

Mother-to-child transmission of chikungunya virus was reported during the 2005–2006 outbreak on Réunion Island, France. To determine the effects of this virus on pregnancy outcomes, we conducted a study of pregnant women in Réunion in 2006. The study population was composed of 1,400 pregnant women (628 uninfected, 658 infected during pregnancy, 27 infected before pregnancy, and 87 infected on unknown dates). We compared pregnancy outcomes for 655 (628 + 27) women not infected during pregnancy with 658 who were infected during pregnancy. Infection occurred during the first trimester for 15% of the infected women, the second for 59%, and the third for 26%. Only hospital admission during pregnancy differed between infected and uninfected women (40% vs. 29%). Other outcomes (cesarean deliveries, obstetric hemorrhaging, preterm births, stillbirths after 22 weeks, birthweight, congenital malformations, and newborn admissions) were similar. This virus had no observable effect on pregnancy outcomes.

## Abstract 2

Except for prenatal hospital admissions for those infected, this virus had no effect on outcomes.

Chikungunya virus infection is transmitted by mosquitoes of the genus *Aedes*. The virus was first isolated in 1952 and is found in eastern Africa, India, and Southeast Asia. Symptoms of infection are high fever and disabling muscle and joint pain, often associated with a rash and mild bleeding. Persons infected usually recover spontaneously in several days to a week (*1*). Fever and arthralgia may occur for several months or even years (*2*). Patients are treated only for their symptoms because there is no specific treatment for the underlying infection (*3*). Before the recent outbreak on the island of Réunion, the disease was not considered life-threatening.

Réunion, a French territory in the southwestern Indian Ocean, has a population of ≈785,000 inhabitants. Medical facilities in Réunion are similar to those in mainland France and other industrialized countries. A major chikungunya outbreak occurred in Réunion in 2005–2006. At the end of this outbreak, seroprevalence was estimated to be 38.2% (95% confidence interval [CI] 35.9%–40.6%); 300,000 (95% CI 283,000–320,000) persons were infected (*4*,*5*). *Aedes albopictus* mosquitoes were the primary vector in this outbreak.

The outbreak began in eastern Africa (*6*). It reached Réunion in March 2005 but was relatively inactive, with only several thousand cases until November 2005, when its incidence unexpectedly increased during summer in the Southern Hemisphere, peaking at 47,000 cases/week during week 5 of 2006. The most recent cases were reported in August 2006. Comparisons of 2006 with previous years showed that mortality rates increased during February, March, and April 2006 (*7*,*8*). Since 2006, the virus has caused several epidemics in the Indian Ocean region (Madagascar, India, Sri Lanka, Thailand, Malaysia, and Singapore). Three new cases of chikungunya were reported in August 2009 on Réunion Island (*9*).

The first cases of virus transmission from mother to child at birth were identified in February 2006; a total of 38 such cases were reported (*10*,*11*). The virus was also found in specimens from 3 early second trimester miscarriages (*12*). When this outbreak began, little information was available about the risk for chikungunya virus infection in pregnant women. In addition to virus transmission at birth, potential complications include transplacental transmission before birth, congenital malformations, stillbirths, growth restriction, and preterm delivery. Chikungunya virus belongs to the same family of viruses (*Togaviridae*) as rubella virus, for which some of these complications have been described (*13*). The high fever that characterizes chikungunya infection could cause uterine contractions or fetal heart rate abnormalities, which might promote spontaneous or induced preterm delivery (cesarean for fetal salvage). The hemorrhagic syndrome described at the onset of infection might be manifested by vaginal bleeding during pregnancy or third-stage hemorrhaging, as reported for infection with dengue virus (*14*,*15*). The proportion of symptomatic and asymptomatic infections was also unknown.

The purpose of our study (the Chikungunya-Mère-Enfant cohort study) was to determine the consequences of chikungunya infection on pregnancy outcomes. These results will be useful to public health officials and physicians who provide care for pregnant women or newborns because chikungunya can be imported by international travelers and the location of *Ae*. *albopictus* mosquitoes has extended beyond the tropics (*16*). These mosquitoes are found in 26 states in the United States and several countries in Europe, where outbreaks are possible (*17*,*18*).

## Methods

We began our study in early April 2006, by planning to recruit all pregnant women (with or without symptoms of chikungunya infection) who received care at 1 of the 6 main maternity units in Réunion. These 6 units accounted for 78% of 14,077 live births in Réunion in 2006. Inclusion in the study was proposed regardless of the reason for a visit or admission. We had planned to include 3,600 women so that sufficient children with in utero chikungunya infection were available to study their psychomotor development. To show a difference of 10 points in the developmental quotient at 24 months of age, it would have been necessary to observe 19 children infected in utero. However, because of the decrease in the outbreak after June 1, we revised our sample size and included only pregnant women who reported clinical signs suggestive of this infection. The study cohort was composed of 1,400 pregnant women (mean term 32 weeks); 1,384 (99%) gave birth in 1 of the 6 participating maternity units. Information on pregnancy outcome for 16 women lost to follow-up was obtained by contacting each one directly. A total of 914 participants were included in April, 386 in May, 88 in June, 5 in July, 2 in August, 4 in September, and 1 in November. In an ancillary study, for 3 days in May 2006, all women who gave birth in the 6 participating units were interviewed to determine how women in the study cohort differed from those not in the study in terms of chikungunya symptoms, parity, age, gestational age of the infant at birth, and mode of delivery.

Serologic status for chikungunya virus infection was determined at participant’s inclusion in the study. All reports of chikungunya fever were confirmed by using serologic testing or detection of the viral genome in any specimen by using real-time reverse transcription–PCR (RT-PCR) (*19*,*20*). Serologic tests with negative results at inclusion were repeated at delivery or when symptoms suggestive of infection appeared. Histologic examinations were performed on placentas of all women who had chikungunya infection during pregnancy. RT-PCR was also performed for placenta and amniotic fluid samples from women with symptoms at delivery.

Date of infection was determined by checking patient history of symptoms or by RT-PCR when available. Women were classified into 2 groups: those infected by chikungunya virus during pregnancy (symptoms during pregnancy confirmed by positive serologic or RT-PCR results) and those not infected (negative serologic results at delivery or during the preceding 7 days). Women infected before pregnancy were considered not infected during pregnancy. We excluded women who were infected but asymptomatic, those whose symptoms could not be dated, and those with inconclusive serologic results from analysis.

We analyzed how women infected by chikungunya virus during pregnancy (658) differed from those who were not infected (655) for general characteristics (age, educational level, marital status, and body mass index), medical history (diabetes and hypertension), and obstetric history (previous pregnancies, history of preterm delivery, small-for-gestational-age, or stillbirths). We then compared pregnancy outcomes (prenatal hospital admission for any reason and for chikungunya symptoms, vaginal bleeding during pregnancy, mode of delivery, obstetric hemorrhage, stillbirth, preterm birth, birthweight, congenital malformations, and newborn hospitalization) between the 2 groups. Obstetric hemorrhage was defined as blood loss >500 mL. We considered only fetal malformations recognized by European Surveillance of Congenital Abnormalities (EUROCAT) (www.eurocat.ulster.antibodies.uk). All malformations recorded were verified by checking either pediatric files or the Réunion congenital anomalies registry, which is affiliated with EUROCAT.

Bivariate analysis of pregnancy outcomes compared means (by Wilcoxon rank-sum test) and percentages (χ^2^ or Fisher exact tests). For multivariate analysis, we adjusted for center, maternal age, educational level, and body mass index. Logistic regression was used to estimate the adjusted odds ratios (ORs). A p value <0.05 was considered significant. Sensitivity analyses were performed to determine whether results changed when either the 27 infected before pregnancy or the 100 women included in the study after May 2006 were omitted from the analysis. Statistical analysis was performed by using SAS version 9.1 software (SAS Inc., Cary, NC, USA).

This prospective multicenter study was reviewed and approved by the ethics committee (Comité de Protection des Personnes) of Tours (no. 2006–2007). It was reported to the French Data Protection Authority (Commission Nationale de l’Informatique et des Libertés).

## Results

Of 1,400 pregnant women included in the study, 705 (50%) reported chikungunya symptoms during pregnancy, 668 (48%) reported no symptoms, and 27 (2%) reported symptoms before pregnancy (Table 1). Specific serologic or RT-PCR tests confirmed the diagnosis of chikungunya infection for 658 (93%) of 705 who reported symptoms during pregnancy. In 6 cases (1%), serologic results for immunoglobulin (Ig) G were negative at delivery, which ruled out infection. Conclusions could not be reached for 41 women (6%) because of missing or inconclusive laboratory data. Negative serologic findings for IgG confirmed the absence of chikungunya infection in 622 (93%) of 668 women with no reported symptoms during pregnancy. Findings were positive for 46 women (7%); these women were considered asymptomatically infected at an unknown date and excluded from the analysis. Chikungunya infection was confirmed for all 27 women with symptoms before pregnancy. Overall, 658 women were classified as infected by chikungunya virus during pregnancy (exposed) and 655 as not infected during pregnancy (not exposed).

**Table 1:** Chikungunya virus infections in 1,400 pregnant women, by onset or lack of symptoms, Réunion, France, 2006*

Symptoms	No. infected	Diagnosis
Symptoms during pregnancy, n = 705		
Yes	658	Exposed
No	6	Not exposed
Unknown	41	Excluded
No symptoms, n = 668		
Yes	46	Excluded
No	622	Not exposed
Symptoms before pregnancy, n = 27		
Yes	27	Not exposed

*Infection was confirmed by positive serologic or reverse transcription–PCR results. Women infected before pregnancy were considered not infected during pregnancy.

Among the 658 exposed women, infection occurred during the first trimester for 99 (15%) women, the second for 387 (59%), and the third for 172 (26%). Infection occurred during the first quarter of 2006 for 536 (81%), before that for 62 (9.4%), and after that for 60 (9.1%). Maternal signs and symptoms were fever (408 cases, 62%), arthralgia (615 cases, 93%), headache (354 cases, 54%), edema (355 cases, 54%), diarrhea (78 cases, 12%), aphthae (63 cases, 9.6%), epistaxis or gingivorrhagia (59 cases, 9.0%), and rash (496 cases, 76%). Overall, 137 (21%) were hospitalized for chikungunya infection for a median duration of 2 days (range 1–75 days). Signs of infection began a median of 108 days before delivery (range 1–263 days), and only 4 infected women (0.6%) had symptoms in the 7 days before delivery.

Pregnancy outcomes included 656 live births to women who were infected and 653 to those who were not infected (including 8 and 14 pairs of twins, respectively); 5 and 8 stillbirths, respectively, after 22 weeks of gestation; and 5 and 8 miscarriages, respectively, before 22 weeks. Of the 4 children born to mothers infected by chikungunya during the last week of pregnancy, 1 newborn had signs of infection on the third day of life, and RT-PCR and IgM serologic analysis confirmed the infection. The mother had had chikungunya symptoms the day before delivery. The other 3 neonates remained asymptomatic and had no detectable IgM against chikungunya virus. Of 624 placentas examined from women found to be infected during pregnancy, only the placenta from the case of mother-to-child transmission had histologic signs compatible with viral infection.

RT-PCR was performed to test for the viral genome in the placenta or amniotic fluid from 3 of the 5 stillbirth fetuses (>22 weeks) of women with chikungunya infections. The test result was positive in 2 cases, in which chikungunya symptoms in the mothers had begun 25 and 70 days before the fetal loss. For the 8 miscarriages before 22 weeks, RT-PCR was performed on trophoblast tissue for 1 case and the result was negative.

Women infected by chikungunya during pregnancy were more likely to have been born in Réunion, to have stopped going to school at a younger age, to be unmarried, overweight, or already have children (Table 2). They also differed by maternity center. Multivariate analysis showed that only 2 characteristics were significantly different: educational level (primary school OR 1.48, 95% CI 1.11–1.97; high school as reference; university OR 0.54, 95% CI 0.38–0.77) and being overweight (body mass index >25 kg/m^2^, OR 1.76, 95% CI 1.22–2.55).

**Table 2:** Characteristics of women infected and not infected with chikungunya virus during pregnancy, Réunion, France, 2006*

Characteristic	Infected, no. (%), n = 658	Not infected, no. (%), n = 655	p value†
Born in Réunion			
Yes	545 (84.1)	510 (79.2)	
No	103 (15.9)	134 (20.8)	0.02
Education			
Primary school	331 (52.2)	214 (34.3	<0.0001
High school	198 (31.2)	200 (32.1)	
University	105 (16.6)	209 (33.6)	
Marital status			
Lives alone	252 (39.0)	207 (32.0)	0.008
Lives with partner	394 (61.0)	440 (68.0)	
History of diabetes			
Yes	17 (2.6)	14 (2.1)	
No	641 (97.4)	641 (97.9)	0.59
History of hypertension			
Yes	23 (3.5)	27 (4.1)	
No	635 (96.5)	627 (95.9)	0.55
Previous pregnancies <22 wks			
Yes (>1)	273 (41.6)	258 (39.5)	
No	384 (58.4)	395 (60.5)	0.45
Mean parity	1.4 (1.6)	1.1 (1.4)	<0.0001
0	216 (32.9)	278 (42.7)	0.0004
1	199 (30.3)	181 (27.8)	
2	110 (16.8)	106 (16.3)	
>3	131 (20.0)	86 (3.2)	
Previous stillbirth or neonatal death			
Yes	22 (3.3)	12 (1.8)	
No	636 (96.7)	643 (98.2)	0.08
Previous preterm delivery			
Yes	44 (6.7)	27 (4.1)	
No	614 (93.3)	626 (95.9)	0.04
Previous child >2,500 g			
Yes	70 (10.7)	55 (8.4)	
No	587 (89.3)	598 (91.6)	0.17
Previous cesarean			
Yes	71 (10.8)	66 (10.1)	
No	587 (89.2)	586 (89.9)	0.69
Mean age at delivery, y	28.6 (6.9)	28.8 (6.4)	0.52
<20	71 (10.8)	69 (10.5)	0.94
20–29	309 (47.0)	303 (46.3)	
>30	278 (42.2)	283 (43.2)	
Mean body mass index, kg/m^2^	24.7 (5.9)	23.4 (5.1)	<0.0001
<25	390 (60.8)	454 (71.5)	<0.0001
25–29	137 (21.3)	113 (17.8)	
>30	115 (17.9)	68 (10.7)	
Center			
1	165 (25.1)	188 (28.7)	<0.0001
2	196 (29.8)	153 (23.4)	
3	62 (9.4)	71 (10.8)	
4	21 (3.2)	9 (1.4)	
5	118 (17.9)	182 (27.8)	
6	96 (14.6)	52 (7.9)	

*Women infected before pregnancy were considered not infected during pregnancy. †By Wilcoxon rank-sum test for continuous variables and χ^2^ test for nominal variables.

After we controlled for potential confounders, the only difference in pregnancy characteristics between infected and uninfected women (Table 3) was the frequency of hospital admissions during pregnancy (40% vs. 29%). This difference disappeared when hospital admission for suspected chikungunya was excluded (26% vs. 28%). Other maternal and neonatal outcomes were similar in both groups. Excluding women infected before pregnancy or included after May 2006 from the analysis did not modify the results (Table 3). Congenital malformations observed in newborns as a function of maternal exposure are shown in Table 4.

**Table 3:** Pregnancy outcome according to chikungunya virus infection during pregnancy, Réunion, France, 2006*

Characteristic	Infected,† no. (%), n = 658	Not infected,‡ no. (%), n = 655	p value	Unadjusted OR (95% CI)	Adjusted OR (95% CI)
Hospital admission during pregnancy					
Yes	266 (40.4)	191 (29.2)		1.65 (1.31–2.07)	1.52 (1.18–1.95)
No	392 (59.6)	464 (70.8)	<0.0001	1	1
Hospital admission during pregnancy, suspected infection with chikungunya virus excluded					
Yes	180 (28.0)	136 (26.1)		0.91 (0.70–1.18)	0.83 (0.62–1.10)
No	464 (72.0)	385 (73.9)	0.48	1	1
Vaginal bleeding during pregnancy					
Yes	55 (8.4)	68 (10.4)		0.79 (0.55–1.15)	0.94 (0.63–1.42)
No	596 (91.6)	584 (89.6)	0.22	1	1
Obstetric hemorrhaging					
Yes	36 (5.6)	42 (6.5)		0.85 (0.54–1.35)	0.87 (0.53–1.42)
No	609 (94.4)	605 (93.5)	0.49	1	1
Mode of delivery§					
Vaginal	545 (83.8)	530 (81.5)	0.27	1	1
Cesarean	105 (16.2)	120 (18.5)		0.85 (0.64–1.14)	0.77 (0.56–1.06)
Mean gestational age, wk§	39.0 (2.1)	38.9 (2.5)	0.55		
<32	8 (1.2)	15 (2.3)	0.26	0.52 (0.22-1.24)	0.48 (0.19–1.23)
32–36	53 (8.2)	60 (9.2)		0.86 (0.59–1.27)	0.78 (0.51–1.20)
>37	589 (90.6)	575 (88.5)		1	1
Mean birthweight, g§	3,116 (549)	3.056 (620)	0.27		
<2,000	20 (3.1)	32 (4.9)	0.36	0.62 (0.35–1.11)	0.66 (0.36–1.22)
2,000–2,999	235 (35.9)	236 (35.7)		0.99 (0.79–1.25)	1.01 (0.79–1.30)
3,000–3,999	372 (56.9)	371 (56.1)		1	1
>4,000	27 (4.1)	22 (3.3)		1.22 (0.69–2.19)	1.25 (0.65–2.39)
Stillbirth after 22 wk§					
Yes	5 (0.8)	8 (1.2)		0.63 (0.20–1.93)	0.61 (0.18–2.07)
No	653 (99.2)	656 (98.8)	0.41	1	1
Congenital malformation					
Yes	19 (2.9)	15 (2.2)		1.36 (0.68–2.74)	1.54 (0.68–3.49)
No	647 (97.1)	654 (97.8)	0.48	1	1
Admission to neonatal care§					
Yes	53 (8.1)	55 (8.3)		0.97 (0.65–1.44)	1.03 (0.67–1.58)
No	605 (91.9)	609 (91.7)	0.88	1	1

*OR, odds ratio; CI, confidence interval. OR was adjusted for center, educational level, body mass index, and maternal age. Women infected before pregnancy were considered not infected during pregnancy. †Of the 658 women who were infected, 650 had delivered a child after 22 weeks; 658 children were delivered by these women. ‡Of the 655 women who were not infected, 650 had delivered a child after 22 weeks; 664 children were delivered by these women. §Miscarriage before 22 weeks was excluded.

**Table 4:** Congenital malformation classification, according to ICD-10 code, as a function of maternal exposure to chikungunya virus during pregnancy, Réunion, France, 2006*

Classification	Exposure to chikungunya virus during pregnancy, no. newborns, n = 34	
	Yes	No
Chromosomal (Q90, Q91, Q96)	3	1
Neural tube (Q03, Q05)	3	0
Cardiovascular (Q20, Q21, Q25, Q26)	5	1
Kidneys, urinary tract, genital organs (Q53, Q55, Q61, Q62, Q63)	1	5
Limbs, thorax, bones, and spine (Q66, Q69, Q71, Q74, Q76)	5	9
Ear, cleft palate (Q17, Q35)	3	0
Other (D22, Q33, Q40, Q42, Q89, T21)	4	3

*ICD-10, International Classification of Diseases, 10th revision. Total exceeds 34 because 1 child had 3 types of malformations and 7 children had 2 types.

In early May, we conducted a 3-day survey of all women giving birth in the maternity units participating in the study. Of 113 women interviewed, 43% (49) were included in the study cohort. The inclusion rate differed according to maternity unit, ranging from 16% to 88%. The mean proportion of women asked to participate was 62% (70/113), and the mean acceptance rate was 70% (49/70); 43% (21) of the women included thought that they had had chikungunya infection during pregnancy compared with 6% (4) of those not included (p<0.0001). Mean parity (2.1 vs. 2.6; p = 0.08), mean maternal age (28.6 years vs. 29.1 years; p = 0.70), mean gestational age at delivery (39.1 weeks vs. 38.7 weeks; p = 0.14), and mode of delivery (18% vaginal vs. 17% cesarean; p = 0.87) did not differ between the women who were or were not included.

## Discussion

In this comparative study, we did not observe any effect of chikungynya infection on pregnancy outcomes except for the number of prenatal maternal hospital admissions for chikungunya symptoms. Our study involved a high proportion of maternity units and births in Réunion. Women included in the study in April 2006 accounted for 73% (905/1,240) of all live births in Réunion. Systematic determination of serologic status by identification of specific IgM and IgG confirmed infection status. All patients for whom chikungunya infection during pregnancy was uncertain were excluded. We excluded women who had positive serologic results but did not report symptoms or have a positive RT-PCR result because we could not identify the date of infection. Studies during the outbreak in Réunion showed that IgM tended to persist for 12 to 24 months and cannot be used to identify the date of infection (*21*).

Because inclusion in the study began in April 2006 after the outbreak had peaked, we could not analyze pregnancies completed before this date. Therefore, our study does not describe the consequences of the outbreak on the risk for miscarriage or preterm delivery during the first quarter of 2006. The study included only pregnancies with outcomes after that quarter. Most of the women were infected before their inclusion. The fact that many women seen in May had already been included at a previous visit in April explains why there were fewer inclusions in May; only pregnant women seen for the first time or who for some reason had not been included in April were eligible. A disadvantage of conducting a study during an outbreak is that its duration cannot be known in advance. For this reason, the number of women was smaller than planned.

Date of infection was estimated by recording the time of symptoms and confirmed by RT-PCR and serologic testing. The positive predictive value of symptoms was reliable because infection was confirmed in ≈93% of women with suggestive symptoms and ruled out in <1% of these women. The negative predictive value was also reliable because serologic results were negative for 93% of the women without symptoms. These values are similar to the positive predictive value (91%) and negative predictive value (87%) of symptoms observed in a survey of a representative sample of the population in Réunion at the end of 2006 (*4*). These results confirm that clinical signs of chikungunya have an excellent predictive value during an outbreak.

Women who thought that they had had chikungunya infection during their pregnancy because they had symptoms were more likely to agree to participate in the cohort than the women without such symptoms. There were also disparities in the inclusion rate according to maternity center. Because of these differences, women included in this study were not representative of the population of pregnant women during this period in Réunion. These differences in the inclusion rate according to symptoms and hospital make it impossible to estimate the attack rate of infection among the population of pregnant women. However, because other characteristics (parity, age, gestational age at delivery, mode of delivery) were similar, sampling did not create any bias for comparisons between exposed and unexposed women.

The rarity of placental histologic lesions (in only 1 of 624 women with chikungunya infection during pregnancy) confirmed the absence of placental infection by the virus and explained the rarity of cases of fetal chikungunya infection before birth (*22*). Couderc et al. recently showed that human syncytiotrophoblast tissue is refractory to chikungunya infection (*23*). During the outbreak in Réunion, only 3 cases of fetal chikungunya infection at the beginning of the second trimester were reported (*12*). All other reported cases involved symptomatic newborns with chikungunya infection in the days after birth, for whom the presumed mechanism of viral transmission was direct passage from maternal blood into the fetal circulation through placental breaches during labor (*11*). Kwiek and others showed that maternal–fetal microtransfusions that occur during labor promote HIV-1 transmission from mother to child (*24*).

Our results are consistent with those of Gérardin et al., who showed that most cases of maternal–fetal transmission of chikungunya virus occurred at birth (*22*). Because we systematically determined chikungunya serologic status, we could compare pregnancy outcomes between infected and uninfected women. We found no difference in risk for hospitalization (except for suspected chikungunya), preterm delivery, low birthweight, or admission to neonatal care. However, the number of women tested enabled us to show a difference of 7% for prevalence of admission during pregnancy, 5% for preterm delivery, 82 g for fetal weight, and 5% for admission to neonatal care (β = 0.20 and α = 0.05).

Stillbirths were not more frequent among women with chikungunya infection during pregnancy than among uninfected women, even though >62% of infected women had fevers. This observation appears to conflict with the hypothesis that fever plays a direct role in in utero deaths. However, because of the rarity of this event (0.64% in 2002 in Réunion) (*25*), the power of the study is insufficient to justify any definitive conclusion.

In our sample, the minimum detectable difference was 1.8% for stillbirths (0.6% vs. 2.4%, β = 0.20 and α = 0.05). For early fetal loss before 22 weeks, the number of events (13/1,313 women) was lower than the number expected probably because most participants were included after that term. For this reason, we could not analyze outcome and reach a conclusion for this point.

Chikungunya infection can also induce hemorrhagic complications (*11*). Overall, 59 infected mothers reported epistaxis or gingivorrhagia, but these symptoms are frequent in pregnant women. We found no difference in the risk for vaginal bleeding during pregnancy or for third-stage hemorrhage.

We observed more congenital malformations in babies exposed to chikungunya in utero than in unexposed babies (19 vs. 15). However, this difference was not significant and we could not reach a definitive conclusion for this factor because only 99 women in our sample had a chikungunya infection during the first trimester. It would have required 1,340 children in each group to show a doubling of the risk (4% vs. 2%) with a power of 80% (β = 0.20 and α = 0.05). There is no information on long-term consequences of in utero exposure to chikungunya. Some newborns in our cohort were followed up until the age of 2 years. Analyses are underway to assess long-term consequences.

Chikungunya infection was more frequent in women with a lower educational level. That disadvantaged populations are overexposed to transmissible infectious diseases, including dengue and chikungunya, has been shown (*26*,*27*). Therefore, during outbreaks, information and protection for all pregnant women should particularly be emphasized, especially for those whose educational level may result in a lack of basic knowledge about disease prevention. It might be useful to screen these women actively and conduct home visits to verify application of basic antivector measures (destruction of mosquito breeding sites and larval havens around the home, wearing of long-sleeved clothing, and use of repellents appropriate for pregnant women and of mosquito netting).

The chikungunya vector (*Ae*. *albopictus*) is found in Asia, Oceania, North and South America, and Europe. International travel creates the possibility of large-scale epidemics in countries previously considered free of chikungunya (*16*,*28*). An epidemic of chikungunya was observed in a temperate zone (Italy) in 2007 (*18*). Our results will provide information for pregnant women in unimmunized populations during epidemics.
